# Engagement, Retention, and Progression to Type 2 Diabetes: A Retrospective Analysis of the Cluster-Randomised "Let's Prevent Diabetes" Trial

**DOI:** 10.1371/journal.pmed.1002078

**Published:** 2016-07-12

**Authors:** Laura J. Gray, Thomas Yates, Jacqui Troughton, Kamlesh Khunti, Melanie J. Davies, The Let’s Prevent Diabetes Team

**Affiliations:** 1 University of Leicester, Department of Health Sciences, Leicester, United Kingdom; 2 University of Leicester, Diabetes Research Centre, Leicester, United Kingdom; 3 Leicester Diabetes Centre, University Hospitals of Leicester, Leicester, United Kingdom; University of Cambridge, UNITED KINGDOM

## Abstract

**Background:**

Prevention of type 2 diabetes mellitus (T2DM) is a global priority. Let’s Prevent Diabetes is a group-based diabetes prevention programme; it was evaluated in a cluster-randomised trial, in which the primary analysis showed a reduction in T2DM (hazard ratio [HR] 0.74, 95% CI 0.48–1.14, *p* = 0.18). We examined the association of engagement and retention with the Let’s Prevent Diabetes prevention programme and T2DM incidence.

**Methods and Findings:**

We used data from a completed cluster-randomised controlled trial including 43 general practices randomised to receive either standard care or a 6-h group structured education programme with an annual refresher course for 2 y. The primary outcome was progression to T2DM at 3 y. The characteristics of those who attended the initial education session (engagers) versus nonengagers and those who attended all sessions (retainers) versus nonretainers were compared. Risk reduction of progression to T2DM by level of attendance was compared to standard care. Eight hundred and eighty participants were recruited, with 447 to the intervention arm, of which 346 (77.4%) were engagers and 130 (29.1%) were retainers. Retainers and engagers were more likely to be older, leaner, and nonsmokers than nonretainers/nonengagers. Engagers were also more likely to be male and be from less socioeconomically deprived areas than nonengagers. Participants who attended the initial session and at least one refresher session were less likely to develop T2DM compared to those in the control arm (30 people of 248 versus 67 people of 433, HR 0.38 [95% CI 0.24–0.62]). Participants who were retained in the programme were also less likely to develop T2DM compared to those in the control arm (7 people of 130 versus 67 people of 433, HR 0.12 [95% CI 0.05–0.28]). Being retained in the programme was also associated with improvements in glucose, glycated haemoglobin (HbA1c), weight, waist circumference, anxiety, quality of life, and daily step count. Given that the data used are from a clinical trial, those taking part might reflect a more motivated sample than the population, which should be taken into account when interpreting the results.

**Conclusions:**

This study suggests that being retained/engaged in a relatively low-resource, pragmatic diabetes prevention programme for those at high risk is associated with reductions in the progression to T2DM in comparison to those who receive standard care. Nonengagers and nonretainers share similar high-risk traits. Service providers of programmes should focus on reaching these hard-to-reach groups.

**Trial Registration:**

ClinicalTrials.gov ISRCTN80605705

## Abstract 2

Attendance in a lifestyle and eating educational program is associated with a reduced incidence of diabetes Gray and colleagues reveal.

## Author Summary

**Why Was This Study Done?:**

- Type 2 diabetes can be prevented through lifestyle improvement.
- Randomised trials have shown that education programmes that aim to increase healthy eating and physical activity and reduce weight can prevent or delay type 2 diabetes.
- Prevention programmes usually require participants to attend a number of sessions over a period of time.
- We wanted to assess how well such a programme works in people who either choose not to attend or drop out during the programme and to determine if particular types of participants are more likely to drop out than others.

**What Did the Researchers Do and Find?:**

- We used data from 447 participants in the intervention arm and 433 participants from the control arm of a completed randomised trial that assessed a type 2 diabetes prevention education programme called Let’s Prevent Diabetes.
- The Let’s Prevent Diabetes programme has a 6-h core initial session followed by a 3-h refresher session annually for 2 y.
- We compared the number of people who went on to develop type 2 diabetes over a 3-y period by level of attendance.
- We found that those who attended all three sessions had a diabetes incidence rate of 16.86 per 1,000 person years compared to 63.16 per 1,000 person years in those in the standard care group (hazard ratio 0.12 [95% CI 0.05–0.28]) and 57.60 per 1,000 person years when assessing the whole intervention group irrespective of attendance level.
- Those who attended all sessions were older, leaner, and less likely to be smokers than those who did not.

**What Do These Findings Mean?:**

- We have shown that the success of the diabetes prevention programme was associated with the level of adherence to the programme.
- Those implementing such programmes should consider how to keep participants motivated.
- The main limitation of this work is that the data used were observational and are from a clinical trial and therefore may not be reflective of what would happen in a real-world situation.

## Introduction

The prevention of diabetes is a global health care priority. A recent study estimated that in 2015 there were 5 million people with nondiabetic hyperglycaemia (NDH, also known as “prediabetes” or at high risk of diabetes) in England, equating to around 11.4% of the population aged 16 y and over [1]. A systematic review of progression rates suggested an incidence rate of progression to type 2 diabetes mellitus (T2DM) of 35.6 per 1,000 person years [2]. With rising levels of obesity, sedentary lifestyles, and low fitness, these numbers are expected to rise over the coming decades; the International Diabetes Federation estimates that worldwide levels of NDH will increase from 318 million to 482 million by 2040 [3].

There is robust evidence that T2DM can be prevented or delayed in those with NDH. Pivotal trials conducted globally showed that lifestyle modification programmes promoting a healthy diet, weight loss, and increased physical activity could reduce the incidence of T2DM by up to 58% [4–7]. These programmes were intensive—for example, in the first year of the United States Diabetes Prevention Programme (DPP), participants received 16 1-h one-to-one counselling sessions followed by an average of eight additional contacts and two telephone consultations [4,8]. Participants were also offered supervised exercise classes. The difficulty, therefore, has been translating such programmes in a resource-limited setting, such as the National Health Service (NHS). A review of programmes that had attempted to translate these findings into a real-world setting found a lower T2DM reduction of 26% [9].

The Let’s Prevent Diabetes programme is a 6-h structured group education programme with annual 3-h refresher sessions and telephone support that aims to translate the findings of the large-scale prevention studies into a pragmatic lower-resource programme suitable for delivery in the NHS [10,11]. The programme was evaluated in a published 3-y cluster-randomised trial, in which the primary analysis showed a no reduction in T2DM (hazard ratio [HR 0.74], 95% CI 0.48–1.14, *p* = 0.18) and modest benefits in biomedical, lifestyle, and psychosocial outcomes in those receiving the Let’s Prevent Diabetes programme compared to those receiving standard care. The per-protocol analysis excluding those participants from the intervention arm who did not attend the initial education session also showed no reduction in T2DM incidence (HR 0.65, 95% CI 0.41–1.03, *p* = 0.07). When assessing the joint distribution of cost and effect differences (measured using quality-adjusted survival), the programme was estimated to be cost-effective at a willingness-to-pay threshold of £20,000 per quality-adjusted life year (QALY) gained [10].

In 2014, the NHS Five Year Forward View outlined an ambition for England to be the first country to implement a national NHS Diabetes Prevention Programme (NDPP) [12]; the programme will launch in 2016. In order to inform the design and implementation of the NDPP, this study aimed to examine the effects of engagement and retention with the Let’s Prevent Diabetes education programme on the outcome found in comparison to standard care. A further aim was to examine the characteristics of those participants who completed the entire programme (retainers) and those who failed to engage with the education at all (nonengagers).

## Methods

The methodology of the Let’s Prevent Diabetes trial and the main trial results have been published previously [11,13,14]. Briefly, the Let’s Prevent Diabetes trial cluster randomised general practices to either the intervention or standard care. Participants found to have NDH via a two-stage risk score screening programme were invited to take part in the trial [14,15]. The inclusion criteria for screening were ages 40–75 y if white European or 25–75 y if South Asian. Participants were excluded if they were unable to give informed consent, pregnant, or lactating, had established diabetes or a terminal illness, or if they required an interpreter for a language other than one of the locally used South Asian languages accommodated within the trial. All those agreeing to take part received an oral glucose tolerance test (OGTT). Only participants who were identified as having NDH (impaired fasting glucose [IFG] and/or impaired glucose tolerance [IGT] WHO 1999 criteria [16]) during screening took part in the randomised controlled trial (RCT). Participants within intervention practices were invited to attend the Let’s Prevent Diabetes programme [17], which is a group-based education programme underpinned by psychological theories aimed at increasing knowledge and promoting realistic perceptions of NDH and by promoting healthy behaviours (reducing weight, following a healthy diet, and increasing physical activity). The programme involves a 6-h session at baseline followed by 3-h refresher sessions at 12 and 24 mo, which reinforced key messages. In addition, participants received a 15-min telephone call every 3 mo from health care professionals trained to offer ongoing support in behaviour change. Those who did not attend the initial session were not invited to the refresher sessions but continued to be followed up. Participants in standard care practices received an information booklet that included information on risk factors for T2DM and how dietary and lifestyle changes and increased physical activity can prevent progression to T2DM.

The primary outcome of the trial was progression to T2DM assessed over 3 y. T2DM was diagnosed according to WHO 1999 criteria/guidelines [16], and from January 2013, HbA1c was also incorporated into the diagnostic criteria [18]. Secondary outcomes included glucose, lipid levels, blood pressure, weight, waist, and body mass index (BMI). Participants also completed a questionnaire containing a number of validated questionnaires that included measures of self-reported sitting time [19], anxiety and depression [20], and quality of life [21]. Participants also wore a sealed pedometer (NL-800, New Lifestyles, Lees Summit, Missouri, US) with a 7-d memory during waking hours to provide habitual ambulatory activity (average daily step count was derived by summing total accumulated steps and dividing by days worn). Outcomes were assessed at baseline and 6, 12, 24, and 36 mo post baseline. Follow-up rates were similar across the two intervention groups [11].

### Statistical Analysis

Here we present the results from a secondary analysis of the intervention arm of a completed randomised trial. This analysis was not part of the original statistical analysis plan for the trial, and a separate prospective analysis plan for the analyses presented here was not written prior to undertaking the work; therefore, these results should be viewed as hypothesis generating. The baseline characteristics of participants in the intervention arm grouped by level of attendance were compared; we compared those who attended the first education session with those who did not (these are termed engagers versus nonengagers throughout) and those who attended all education sessions with those who did not (these are termed retainers versus nonretainers throughout). This analysis differs from the per-protocol analysis published as part of the primary trial findings as that analysis restricted the intervention group to those who attended at least the first education session compared to standard care [11]. Logistic regression was used to compare groups; standard errors were adjusted for the clustering.

Progression to T2DM was analysed by education attendance level. Participants not developing T2DM were censored at the date of their last clinical appointment. Cox proportional hazards models with the intervention group as a covariable were fitted; practices were assumed to have the same frailty. Secondary outcomes at 3 y were compared between (1) retainers versus standard care and (2) retainers versus nonretainers. For the secondary outcomes assessed, participants who developed T2DM during the study had their last value from before their diagnosis carried forward for the remainder of the study. This method was used in a previous similar study [22]. Secondary outcomes were analysed using generalised estimating equation models with an exchangeable correlation structure, which adjusted for clustering [23]. We also adjusted both analyses (primary and secondary outcomes) by age, sex, deprivation score, smoking status, and BMI; both adjusted and unadjusted data are shown.

Given that 55% of the intervention participants attended the core session and at least one refresher session compared to only 29% who attended all sessions, we conducted a number of sensitivity analyses that repeated the retainer analyses using the 55% as the group of interest rather than the 29% who attended all sessions. We have defined this group as “plus min one”.

Statistical significance was set at 5% for all analyses, with 95% confidence intervals reported. Throughout, missing data were not replaced, and an available case approach was taken. All analyses were conducted using Stata version 13.

## Results

**Fig 1:**
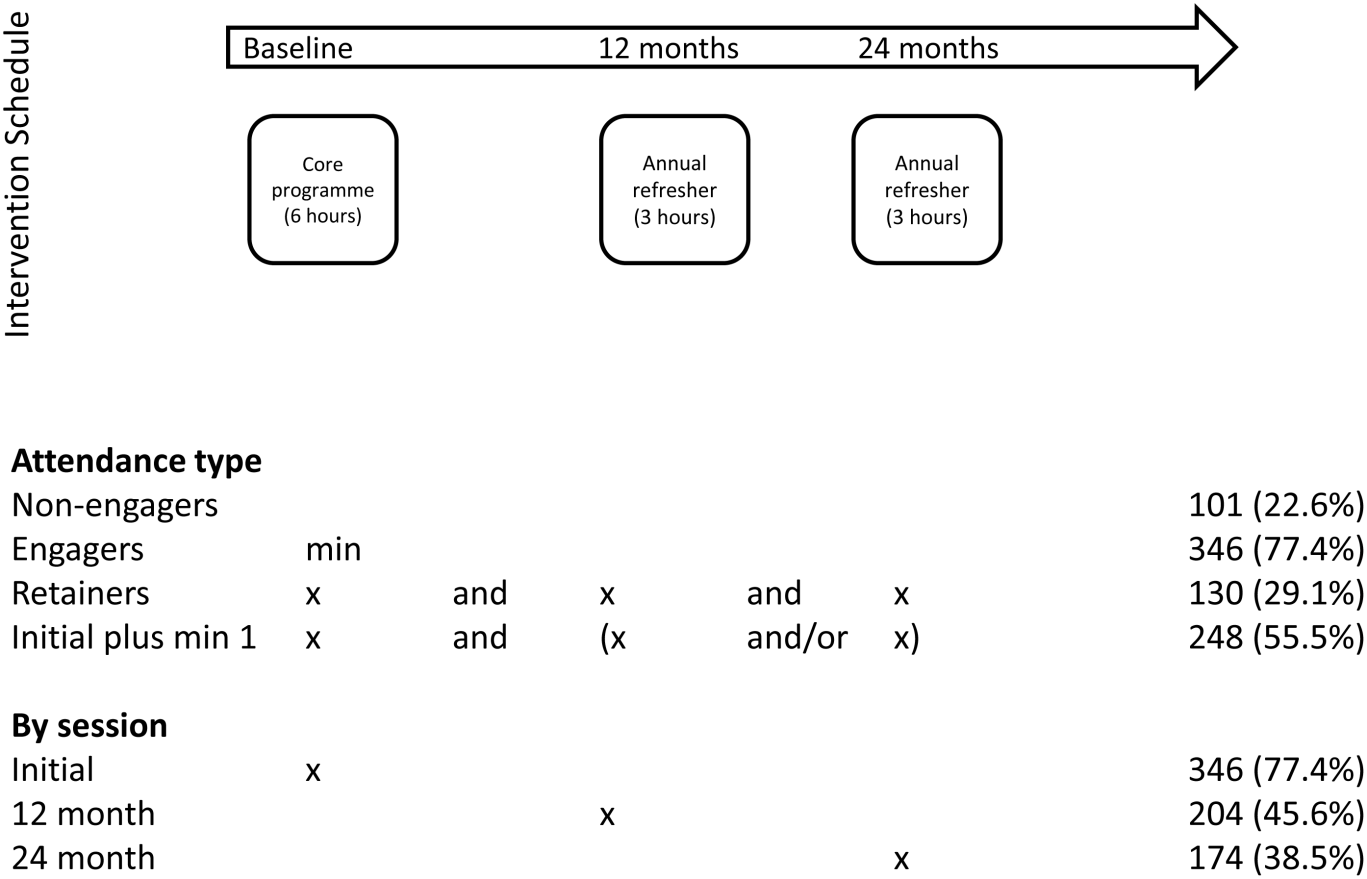
Attendance at education sessions.

In total, 880 participants were recruited from 43 general practices, 447 to intervention practices and 433 to control practices [11]. Of the participants included from practices randomised to the intervention group, 346 (77.4%) attended the initial 6-h education session, i.e., were engagers (Fig 1). One hundred and thirty participants (29.1%) attended all sessions—i.e., were retainers, with 248 attending the initial sessions plus a minimum of one refresher session (55.5%). The baseline characteristics across levels of attendance are given in S1 Table.

Table 1 compares the baseline characteristics of engagers versus nonengagers. Overall, engagers were older and more likely to be male and nonsmokers, be from less socioeconomically deprived areas, and have lower BMI than nonengagers. Table 2 compares retainers against nonretainers. Retainers were more likely to be older, nonsmokers, and have a lower BMI than nonretainers. Similar finding were found when assessing those who attended at least one refresher session (S2 Table).

**Table 1:** Comparison of baseline characteristics of those who engage versus nonengagers (defined as attending the first education session). Data are given as mean (standard deviation [SD]) unless otherwise stated. The odds ratio gives the odds associated with being an engager compared to a nonengager, 95% CI adjusted for clustering.

	Nonengagers	Engagers	Odds Ratio (95% CI)	*P*-value
Number of Participants	101 (22.6)	346 (77.4)		
Age	62.5 (9.1)	64.3 (7.1)	1.03 (1.01–1.06)	0.02
Male *n* (%)	52 (51.5)	230 (66.5)	1.87 (1.12–3.11)	0.02
White European, *n* (%)	81 (80.2)	296 (85.8)	1.49 (0.78–2.84)	0.22
Deprivation, Median (IQR)	17.8 (10.4, 36.5)	12.1 (7.0, 23.6)	0.97 (0.96–0.99)	<0.001
Current Smoker, *n* (%)	22 (21.8)	16 (4.6)	0.17 (0.09–0.32)	<0.001
Prescribed Statins, *n* (%)	37 (38.5)	149 (45.9)	1.35 (0.86–2.14)	0.19
Prescribed Antihypertensives, *n* (%)	60 (59.4)	215 (62.1)	1.12 (0.68–1.86)	0.66
History CVD, *n* (%)	18 (17.8)	57 (16.5)	0.91 (0.46–1.79)	0.78
HbA1c (%)	6.2 (0.4)	6.1 (0.4)	0.62 (0.38–0.99)	0.05
HbA1c (mmol/mol)	43.9 (4.9)	43.0 (4.6)	0.96 (0.92–0.99)	0.05
Total Cholesterol (mmol/l)	5.1 (1.0)	5.0 (1.0)	0.90 (0.76–1.07)	0.24
HDL Cholesterol (mmol/l)	1.4 (0.4)	1.4 (0.5)	0.97 (0.64–1.48)	0.89
LDL Cholesterol (mmol/l)	3.0 (0.9)	2.9 (0.9)	0.96 (0.78–1.19)	0.72
Triglycerides (mmol/l)	1.9 (1.0)	1.7 (0.9)	0.79 (0.67–0.94)	0.01
Systolic Blood Pressure (mmHg)	147.3 (24.3)	148.1 (19.5)	1.00 (0.99–1.02)	0.78
Diastolic Blood Pressure (mmHg)	86.0 (13.2)	86.8 (10.2)	1.01 (0.99–1.03)	0.54
Heart Rate (bmp)	70.4 (14.5)	67.7 (12.6)	0.98 (0.97–1.00)	0.06
Weight (kg)	89.4 (17.0)	90.0 (16.5)	1.00 (0.99–1.02)	0.74
BMI (kg/m^2^)	32.8 (5.4)	31.7 (5.2)	0.96 (0.94–0.99)	0.01
Waist Circumference (cm)	107.8 (12.1)	108.1 (12.4)	1.00 (0.98–1.02)	0.83
Average Steps per Day	5,689.2 (2,786.4)	6,260.4 (2,784.4)	1.00 (0.99–1.00)	0.13

Abbreviations: CVD, cardiovascular disease; HDL, high-density lipoprotein; IQR, interquartile range; LDL, low-density lipoprotein

**Table 2:** Comparison of baseline characteristics of those who retain (defined as attending all education sessions) versus nonretainers. Data are given as mean (SD) unless otherwise stated. The odds ratio gives the odds associated with being a retainer compared to a nonretainer, 95% CI adjusted for clustering.

	Nonretainers	Retainers	Odds Ratio (95% CI)	*P*-value
Number of Participants	317 (70.9)	130 (29.1)		
Age	63.3 (7.6)	65.4 (7.4)	1.04 (1.00–1.08)	0.03
Male *n* (%)	190 (59.9)	92 (70.8)	1.62 (0.99–2.66)	0.06
White European, *n* (%)	264 (83.5)	113 (86.9)	1.31 (0.61–2.82)	0.49
Deprivation, Median (IQR)	13.5 (8.4, 24.7)	11.7 (7.1, 23.9)	1.00 (0.99–1.00)	0.10
Current Smoker, *n* (%)	34 (10.7)	4 (3.1)	0.27 (0.11–0.64)	0.003
Prescribed Statins, *n* (%)	124 (41.3)	50 (51.7)	1.52 (0.96–2.41)	0.07
Prescribed Antihypertensives, *n* (%)	195 (61.5)	80 (61.5)	1.00 (0.57–1.77)	0.99
History CVD, *n* (%)	80 (15.8)	25 (19.2)	1.27 (0.68–2.36)	0.45
HbA1c (%)	6.1 (0.4)	6.0 (0.4)	0.62 (0.38–1.03)	0.07
HbA1c (mmol/mol)	43.5 (4.8)	42.5 (4.2)	0.96 (0.91–1.00)	0.07
Total Cholesterol (mmol/l)	5.0 (1.1)	5.0 (0.9)	0.93 (0.78–1.11)	0.44
HDL Cholesterol (mmol/l)	1.4 (0.5)	1.3 (0.4)	0.89 (0.56–1.41)	0.62
LDL Cholesterol (mmol/l)	3.0 (0.9)	3.0 (0.8)	1.00 (0.82–1.21)	0.98
Triglycerides (mmol/l)	1.8 (0.9)	1.7 (0.9)	0.88 (0.68–1.15)	0.35
Systolic Blood Pressure (mmHg)	147.3 (21.2)	149.5 (19.4)	1.01 (0.99–1.02)	0.33
Diastolic Blood Pressure (mmHg)	86.4 (11.2)	87.2 (10.3)	1.01 (0.99–1.02)	0.49
Heart Rate (bmp)	68.7 (12.9)	67.4 (13.5)	0.99 (0.97–1.01)	0.45
Weight (kg)	90.4 (17.3)	88.6 (14.8)	0.99 (0.99–1.00)	0.09
BMI (kg/m^2^)	32.4 (5.4)	31.0 (4.7)	0.95 (0.92–0.98)	<0.001
Waist Circumference (cm)	108.3 (12.7)	107.3 (11.4)	0.99 (0.98–1.00)	0.13
Average Steps per Day	6,159.0 (2,888.8)	6,089.8 (2,564.7)	1.00 (0.99–1.00)	0.82

**Fig 2:**
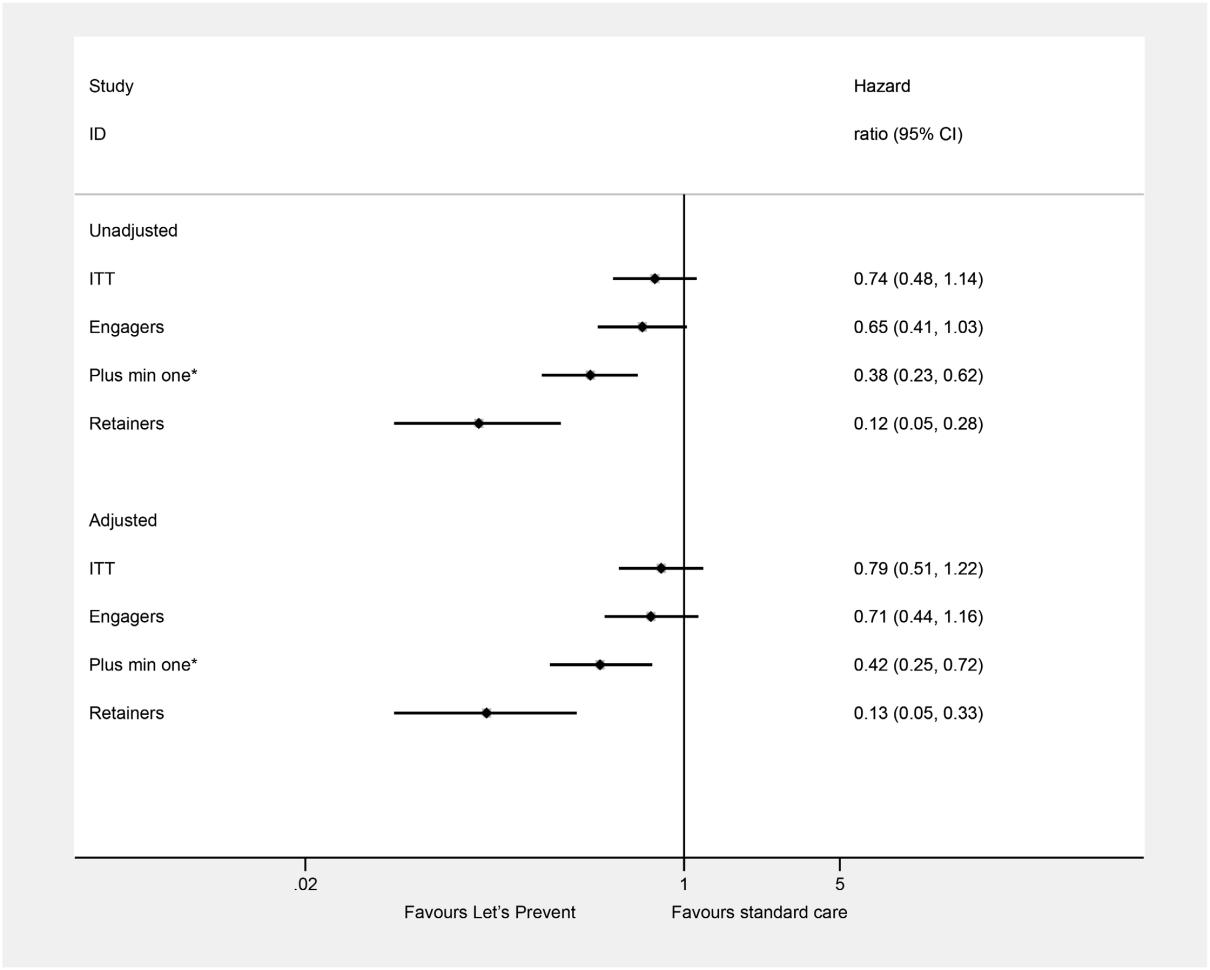
Incidence of type 2 diabetes by attendance. HR (95% CI) takes into account clustering, and the adjusted models include age, sex, deprivation score, smoking status, and BMI. Engagers: attended the initial education session; retainers: attended all education sessions; plus min one: attended the initial education plus a minimum of one refresher session; * these are not mutually exclusive groups.

The intention-to-treat (ITT) primary analysis of the Let’s Prevent Diabetes trial showed no reduction in the incidence of T2DM (Fig 2), an incidence rate of 57.60 per 1,000 person years in the intervention group compared to 63.16 per 1,000 person years in the standard care group (Table 3). When assessing the difference in incidence between the intervention and standard care group by attendance, a dose-response relationship was observed, with a greater reduction in incidence being seen with increasing retention; incidence rate in those who attended all sessions was 16.86 per 1,000 person years. A statistically significant association was observed in those who attend the initial session and then a minimum of one refresher session (HR 0.38 [95% CI 0.236–0.62]) and in retainers (HR 0.12 [95% CI 0.05–0.28]) compared to standard care. Adjusting for age, sex, deprivation score, smoking status, and BMI did not alter the interpretation of these results.

**Table 3:** Comparison of T2DM events and incidence rates across groups.

Group	T2DM Events *n*	Incidence Rate per 1,000 Person Years (95% CI)
Standard Care	67/433	63.16 (49.71–80.24)
Intervention		
ITT	64/447	57.60 (45.09–73.59)
Engagers	51/346	53.04 (40.31–69.80)
Plus Min One	30/248	39.61 (27.70–56.65)
Retainers	7/130	16.86 (8.04–35.36)

ITT, intention to treat

Table 4 shows two subgroup analyses for key secondary outcomes. Retaining was associated with statistically significant improvements in fasting and 2-h glucose and HbA1c compared to those receiving standard care and nonretainers. Retainers had on average HbA1c values that were 0.16% lower than those who received standard care at 3 y; this seemed to be driven by an increase in HbA1c in the standard care group. Retainers were also significantly leaner than those who received standard care and nonretainers, with lower weight, BMI, and waist circumference. Retainers were on average 1.70 kg lighter than nonretainers and 1.28 kg lighter than those in the standard care arm. Lower levels of anxiety and higher quality of life were also seen in those who retained compared to standard care. On average, those who completed the whole programme had a significantly higher step count of 925 steps per d compared to standard care. Adjusting for age, sex, deprivation score, smoking status, and BMI did not alter the interpretation of these results.

**Table 4:** Secondary outcomes in those retaining compared to standard care and retainers versus nonretainers. Coefficients show the mean effect of the intervention compared to standard care adjusted for baseline value and cluster. Adjusted columns are additionally adjusted for age, sex, deprivation score, smoking status, and BMI.

	Mean Change from Baseline to 3 y (SD)			Retainers versus SC		Retainers versus Nonretainers	
	Retainers Only	Nonretainers Only	Standard care	Unadjusted	Adjusted	Unadjusted	Adjusted
Fasting Glucose	−0.05 (0.62)	0.19 (0.83)	0.16 (0.64)	−0.21 (−0.31, -0.10)***	−0.19 (−0.30, −0.09)***	−0.23 (−0.38, −0.08)**	−0.19 (−0.33, −0.05)**
2-h Glucose	−1.51 (2.02)	−0.37 (2.55)	−0.71 (2.45)	−0.84 (−1.21, −0.48)***	−0.71 (−1.08, −0.35)***	−1.21 (−1.68, −0.74)***	−1.15 (−1.58, −0.71)***
Hba1c (%)	−0.14 (0.34)	−0.03 (0.42)	0.01 (0.44)	−0.16 (−0.28, −0.04)*	−0.15 (−0.27, −0.03)*	−0.11 (−0.18, −0.04)**	−0.11 (−0.18, −0.03)**
Total Cholesterol (mmol/l)	−0.29 (0.90)	−0.26 (0.81)	−0.18 (0.90)	−0.14 (−0.32, 0.04)	−0.11 (−0.30, 0.08)	−0.05 (−0.26, 0.16)	−0.02 (−0.21, 0.17)
HDL Cholesterol (mmol/l)	0.05 (0.35)	0.01 (0.42)	0.02 (0.46)	−0.01 (−0.07, 0.07)	−0.02 (−0.09, 0.05)	0.02 (−0.02, 0.07)	0.02 (−0.03, 0.06)
LDL Cholesterol (mmol/l)	−0.36 (0.72)	−0.31 (0.69)	−0.24 (0.78)	−0.12 (−0.25, 0.01)	−0.10 (−0.23, 0.03)	−0.05 (−0.20, 0.10)	−0.02 (−0.16, 0.11)
Triglyceride (mmol/l)	−0.10 (0.76)	−0.02 (0.76)	0.02 (0.80)	−0.12 (−0.29, 0.05)	−0.09 (−0.25, 0.08)	−0.09 (−0.30, 0.11)	−0.07 (−0.26, 0.12)
Body Weight (kg)	−1.55 (4.23)	0.03 (4.71)	−0.46 (5.02)	−1.28 (−2.40, −0.16)*	−1.30 (−2.42, −0.19)*	−1.70 (−2.78, −0.63)**	−1.49 (−2.52, −0.45)**
BMI (kg/m^2^)	−0.47 (1.60)	0.02 (1.68)	−0.17 (1.77)	−0.40 (−0.79, −0.01)	−0.39 (−0.79, 0.01)	−0.53 (−0.87, −0.19)**	−0.46 (−0.80, −0.12)**
Waist Circumference (cm)	−4.98 (5.44)	−2.76 (5.86)	−3.13 (6.32)	−2.06 (−3.41, −0.72)**	−1.89 (−3.13, −0.65)**	−2.24 (−4.06, −0.41)*	−1.92 (−3.53, −0.32)*
Systolic BP (mmHg)	−7.67 (15.72)	−7.51 (17.44)	−8.00 (17.36)	0.86 (−2.97, 4.69)	0.21 (−3.37, 3.79)	−0.20 (−4.10, 3.70)	−0.64 (−4.16, 2.87)
Diastolic BP (mmHg)	−4.31 (10.34)	−3.01 (9.54)	−2.50 (10.92)	−1.08 (−2.86, 0.71)	−1.39 (−3.20, 0.42)	−0.89 (−2.34, 0.55)	−0.65 (−2.11, 0.80)
Heart Rate (bpm)	−1.22 (9.17)	−0.37 (10.22)	−0.63 (10.12)	−1.03 (−2.77, 0.71)	−0.56 (−2.27, 1.15)	−0.74 (−2.80, 1.32)	−0.25 (−2.31, 1.80)
Anxiety Score	−1.03 (2.60)	−0.39 (2.93)	−0.38 (2.65)	−0.54 (−1.05, −0.03)*	−0.61 (−1.15, −0.07)*	−0.72 (−1.30, −0.14)*	−0.76 (−1.31, −0.21)**
Depression Score	−0.22 (2.12)	0.08 (2.53)	0.03 (2.14)	−0.29 (−0.77, 0.19)	−0.34 (−0.81, 0.13)	−0.39 (−0.94, 0.16)	−0.41 (−0.93, 0.10)
Quality of Life	0.003 (0.05)	−0.001 (0.06)	−0.02 (0.06)	0.02 (0.01, 0.04)**	0.02 (0.01, 0.04)**	0.01 (−0.01, 0.02)	0.01 (−0.01, 0.02)
Sitting Time (mins)	−24.63 (164.67)	−38.12 (185.41)	−6.53 (170.27)	−18.22 (−45.10, 8.66)	−18.41 (−44.45, 7.62)	3.37 (−27.47, 34.22)	10.46 (−24.05, 44.97)
Average Steps	180.56 (2,864.29)	−612.04 (2,700.81)	−776.14 (2740.46)	925.45 (236.31, 1,614.59)*	806.91 (124.39, 1,489.44)*	701.70 (−29.33, 1,432.72)	608.85 (7.67, 1,210.05)*

Abbreviations: BP, blood pressure; SC, standard care.

* *p* < 0.05,

** *p* < 0.01,

*** *p* < 0.001

The sensitivity analysis of those who attended at least one refresher session is shown in S3 Table. The majority of the findings are consistent with those based on participants who attended all sessions, although there were some notable differences. Comparing to standard care using this group as the comparator, no differences in change in body weight or average step count were seen. When comparing within the intervention group those who attended at least one refresher session compared to those who did not, larger differences were seen for systolic blood pressure (unadjusted −2.28 mmHg, adjusted −3.21 mmHg) than when assessing retainers. Across both comparisons, no differences in anxiety score were seen.

## Discussion

Although the primary analysis of the Let’s Prevent Diabetes trial including all randomised participants (including those who never accessed the intervention) showed a no effect on the incidence of T2DM, we have shown a large difference in T2DM incidence over 3 y (88%; HR 0.12 [95% CI 0.05–0.28]) in those who attended all education sessions compared to standard care. A dose-response relationship with the incidence of T2DM being reduced as attendance increased was seen. Importantly, these relationships were independent of age, sex, smoking status, deprivation score, and BMI. Significantly improved secondary outcomes were also seen in those who retained compared to those who received standard care and those who did not complete all education sessions. Similar results were seen when assessing those who attended the core session and at least one refresher session. These results combined suggest that both engagement with a prevention programme and retaining that engagement are critical for the effectiveness of diabetes prevention programmes.

Similar findings have been shown for other non-United Kingdom prevention programmes. In the US, a study that translated the 16-session DPP for use in American Indian and Alaska native communities found a statistically significant lower incidence rate of T2DM in the two-thirds who completed all 16 sessions compared to those who did not, with approximately half the number of cases [24]. The retention rate was higher than that seen in the Let’s Prevent Diabetes trial, which may be reflective of the shorter programme duration—all 16 sessions were delivered within 6 mo. A US-based weight management programme found that both weight and diabetes incidence were reduced in a similar manner to the results shown here when comparing retainers to those who did not participate [25]. A meta-analysis of programmes modelled on the DPP showed that with every additional session attended, weight loss increased by 0.26 percentage points [26]. To date, there is no published data assessing compliance with a diabetes prevention programme and outcome in a UK population.

We have also identified similar characteristics in those who do not engage and those who do not retain. These groups were younger, more likely to be smokers, and have a higher BMI than those who do engage/retain. The younger age may be related to employment and availability to attend multiple sessions run during the working week. Prevention programmes in clinical practice should offer sessions at a variety of times and at weekends or in the workplace. Overweight and obesity are major drivers of T2DM risk; additionally, smokers may represent a less healthy cohort. Therefore, developing mechanisms to engage this higher-risk group are essential. Deprivation was also found to be important in terms of initial engagement, with those from more deprived areas being less likely to engage with the education. This is consistent with a 10-wk UK prevention programme that also reported an association between deprivation and follow-up noncompletion [27].

The NDPP will be a 9-mo programme including a minimum of 13 contacts totalling 16 hours, as informed by the evidence review of completed real-world diabetes prevention programmes and National Institute for Health and Care Excellence (NICE) recommendations [9]. The challenge will be replicating these outcomes in a real-world setting and ensuring retention in the programme for 9 mo. Previous studies have shown a dilution of effect when translating programmes that have worked in a research setting to routine clinical practice [28]. Here, we show that even in a much less resource-intensive programme, albeit over a longer duration, adherence was not achieved in the majority of participants. Unfortunately, no data were collected within the Let’s Prevent trial regarding why participants chose not to attend the education sessions. Future studies in this area should aim to collect such data to inform the development of future programmes. Here, we have identified drivers for engaging and retaining; the providers of the NDPP may want to focus some additional resources on these hard-to-engage-and-retain groups, such as those of working age and those from deprived areas. The data collected during the rollout of the NDPP could also be used to assess the minimum dose of intervention required to see reductions in risk factors in a real-world setting.

This study has limitations that should be considering when interpreting the results. These data are from a cluster-randomised trial; therefore, it could be argued that this may not be reflective of what would happen if the Let’s Prevent Diabetes programme was rolled out in the real world. Those taking part might reflect a more engaged sample than the population with NDH; indeed, only 19% of those invited to take part consented [11]. Therefore, these results should be viewed as optimistic. Additionally, this is a secondary unplanned analysis. The Let’s Prevent Diabetes trial was not powered to assess outcomes by attendance status, and the multiple testing may have increased the chance of making a type one error—although, reassuringly, the pattern of results seen here is consistent with other research in this area. The trial was powered to assess the data on an intention-to-treat basis that includes all those randomised irrespective of the intervention received [29]. It could be argued that this is more reflective of the results that could be achieved in clinical practice [30]. However, this type of efficacy analysis is useful for informing future studies and implementation policy that may assess novel methods for increasing retention to achieve such outcomes. Finally, participants also received a 15-min telephone call every 3 mo from health care professionals trained to offer ongoing support in behaviour change. This analysis has not considered the effectiveness of this additional support.

This study suggests characteristics that could be used to identify those at high risk of either not engaging or not retaining. Future studies could build on these findings to develop validated models for predicting nonengagement/nonretainment. Trials could then assess the effectiveness of providing additional incentives or communications in such groups. The rollout of the NDPP could be a platform on which to embed such studies to increase the efficiency of the programme; similar studies have been embedded in RCTs [31].

In conclusion, we have shown that attending all sessions of the Let’s Prevent Diabetes programme is associated with an 88% reduction in T2DM incidence over 3 y compared to those who received standard care in the trial. Those who do not engage or retain in the whole programme tend to be younger and of a higher T2DM risk status. Commissioners of prevention programmes need to ensure programmes are accessible to all and keep participants engaged and motivated to continue.

## Supporting Information

S1 Data Anonymised dataset. (XLSX)

S1 Table Comparison of baseline characteristics across the groups being compared. (DOCX)

S2 Table Comparison of baseline characteristics of those who attended the core programme plus one or more refresher sessions (plus min one group) versus nonengagers and those who attended only the core session. (DOCX)

S3 Table Secondary outcomes of those who attended the core programme plus one or more refresher sessions (plus min one group) versus nonengagers and those who attended only the core session. (DOCX)

S1 STROBE Checklist Strengthening the Reporting of Observational Studies in Epidemiology checklist. (PDF)

## Abbreviations

BMI: body mass index
DPP: diabetes prevention programme
HbA1c: glycated haemoglobin
HR: hazard ratio
IFG: impaired fasting glucose
IGT: impaired glucose tolerance
ITT: intention to treat
NDH: nondiabetic hyperglycaemia
NDPP: NHS Diabetes Prevention Programme
NHS: National Health Service
NICE: National Institute for Health and Care Excellence
OGTT: oral glucose tolerance test
QALY: quality-adjusted life year
RCT: randomised controlled trial
SD: standard deviation
T2DM: type 2 diabetes mellitus
