# Supplementary material for: Engagement, Retention, and Progression to Type 2 Diabetes: A Retrospective Analysis of the Cluster-Randomised "Let's Prevent Diabetes" Trial
Source: PLoS Med. 2016 Jul 12;13(7):e1002078. doi: 10.1371/journal.pmed.1002078 (PMC4942137; doi:10.1371/journal.pmed.1002078)
Supplement: S1 Table — (DOCX) [file pmed.1002078.s002.docx]

**S1 Table Comparison of baseline characteristics across the groups being compared**

Data given as mean (SD) unless otherwise stated.

|  | Standard Care | Number of intervention sessions attended (those groups marked * are mutually exclusive) | | | | | |
| --- | --- | --- | --- | --- | --- | --- | --- |
|  |  | None* | One (core)* | Minimum One | Two (core plus one refresher)* | Core plus minimum one refresher | Three (core plus two refreshers)* |
| Descriptor | Standard Care | Non-engagers | Minimum | Engagers | Plus one | Plus min one | Retainers |
| Number of participants, n (%) | 433 | 101 (22.6) | 98 (21.9) | 346 (77.4) | 118 (26.4) | 248 (55.5) | 130 (29.1) |
| Age | 63.9 (7.9) | 62.5 (9.1) | 63.0 (7.3) | 64.3 (7.1) | 64.3 (6.4) | 64.9 (6.9) | 65.4 (7.4) |
| Male, n (%) | 278 (64.2) | 52 (51.5) | 56 (57.1) | 230 (66.5) | 82 (69.5) | 174 (70.2) | 92 (70.8) |
| White European, n (%) | 363 (84.3) | 81 (80.2) | 85 (86.7) | 296 (85.8) | 98 (83.8) | 211 (85.4) | 113 (86.9) |
| Deprivation, median (IQR) | 10.1 (6.3, 18.1) | 17.8 (10.4, 36.5) | 10.6 (5.6, 22.0) | 12.1 (7.0, 23.6) | 14.2 (8.5, 24.2) | 12.4 (8.3, 24.0) | 11.7 (7.1, 23.9) |
| Current smoker, n (%) | 22 (5.1) | 22 (21.8) | 7 (7.1) | 16 (4.6) | 5 (4.2) | 9 (3.6) | 4 (3.1) |
| Prescribed statins, n (%) | 171 (43.3) | 37 (38.5) | 37 (39.8) | 149 (45.9) | 50 (45.1) | 110 (48.5) | 50 (51.7) |
| Prescribed antihypertensives, n (%) | 270 (62.4) | 60 (59.4) | 62 (63.3) | 215 (62.1) | 73 (61.9) | 153 (61.7) | 80 (61.5) |
| History CVD, n (%) | 78 (18.0) | 18 (17.8) | 14 (14.3) | 57 (16.5) | 18 (15.3) | 43 (17.3) | 25 (19.2) |
| HbA1c (%) | 6.1 (0.4) | 6.2 (0.4) | 6.2 (0.5) | 6.1 (0.4) | 6.1 (0.4) | 6.1 (0.4) | 6.0 (0.4) |
| HbA1c (mmol/mol) | 42.8 (4.6) | 43.9 (4.9) | 43.7 (5.1) | 43.0 (4.6) | 42.8 (4.5) | 42.7 (4.3) | 42.5 (4.2) |
| Total cholesterol (mmol/l) | 5.1 (1.1) | 5.1 (1.0) | 5.1 (1.1) | 5.0 (1.0) | 5.0 (1.1) | 5.0 (1.0) | 5.0 (0.9) |
| HDL cholesterol (mmol/l) | 1.4 (0.5) | 1.4 (0.4) | 1.4 (0.5) | 1.4 (0.5) | 1.4 (0.5) | 1.3 (0.5) | 1.3 (0.4) |
| LDL cholesterol (mmol/l) | 3.0 (0.9) | 3.0 (0.9) | 2.9 (0.9) | 2.9 (0.9) | 3.0 (1.0) | 3.0 (0.9) | 3.0 (0.8) |
| Triglycerides (mmol/l) | 1.7 (1.0) | 1.9 (1.0) | 1.8 (1.0) | 1.7 (0.9) | 1.6 (0.7) | 1.6 (0.8) | 1.7 (0.9) |
| Systolic blood pressure (mmHg) | 147.7 (17.7) | 147.3 (24.3) | 147.1 (21.4) | 148.1 (19.5) | 147.4 (18.1) | 148.5 (18.8) | 149.5 (19.4) |
| Diastolic blood pressure (mmHg) | 86.2 (10.6) | 86.0 (13.2) | 86.7 (10.6) | 86.8 (10.2) | 86.6 (10.0) | 86.9 (10.1) | 87.2 (10.3) |
| Heart rate (bmp) | 69.1 (12.1) | 70.4 (14.5) | 68.9 (12.5) | 67.7 (12.6) | 67.1 (11.8) | 67.3 (12.7) | 67.4 (13.5) |
| Weight (kg) | 94.4 (18.9) | 89.4 (17.0) | 90.6 (17.5) | 90.0 (16.5) | 91.1 (17.4) | 89.8 (16.1) | 88.6 (14.8) |
| BMI (kg/m^2^) | 33.1 (5.8) | 32.8 (5.4) | 32.5 (5.4) | 31.7 (5.2) | 31.9 (5.4) | 31.4 (5.0) | 31.0 (4.7) |
| Waist circumference (cm) | 111.3 (13.2) | 107.8 (12.1) | 108.1 (13.7) | 108.1 (12.4) | 108.9 (12.5) | 108.1 (11.9) | 107.3 (11.4) |
| Average steps per day | 6308.1 (3094.4) | 5689.2 (2786.4) | 6372.5 (3199.0) | 6260.4 (2784.4) | 6364.6 (2679.1) | 6217.6 (2616.0) | 6089.8 (2564.7) |
