# Supplementary material for: Engagement, Retention, and Progression to Type 2 Diabetes: A Retrospective Analysis of the Cluster-Randomised "Let's Prevent Diabetes" Trial
Source: PLoS Med. 2016 Jul 12;13(7):e1002078. doi: 10.1371/journal.pmed.1002078 (PMC4942137; doi:10.1371/journal.pmed.1002078)
Supplement: S2 Table — (DOCX) [file pmed.1002078.s003.docx]

S2 Table Comparison of baseline characteristics of those who attended the core programme plus one or more refresher sessions (plus min one group) versus non-engagers and those who attended only the core session

Data given as mean (SD) unless otherwise stated. Odds ratio gives the odds associated with being a plus min one compared to not, 95% CI adjusted for clustering.

|  | Non – plus min one | Plus min one | Odds ratio (95% CI) | P value |
| --- | --- | --- | --- | --- |
| Number of participants | 199 (44.5) | 248 (55.5) |  |  |
| Age | 62.7 (8.2) | 64.9 (6.9) | 1.04 (1.02, 1.06) | 0.001 |
| Male n (%) | 108 (54.3) | 174 (70.2) | 1.98 (1.40, 2.81) | <0.0001 |
| White European, n (%) | 166 (83.4) | 211 (85.4) | 1.17 (0.75, 1.82) | 0.50 |
| Deprivation, median (IQR) | 13.5 (8.4, 25.3) | 12.4 (8.3, 24.1) | 1.00 (1.00, 1.00) | 0.43 |
| Current smoker, n (%) | 29 (14.6) | 9 (3.6) | 0.22 (0.12, 0.40) | <0.0001 |
| Prescribed statins, n (%) | 74 (39.2) | 110 (48.5) | 1.46 (1.00, 2.14) | 0.05 |
| Prescribed antihypertensives, n (%) | 122 (61.3) | 153 (61.7) | 1.02 (0.64, 1.62) | 0.95 |
| History CVD, n (%) | 32 (16.1) | 43 (17.3) | 1.09 (0.55, 2.18) | 0.80 |
| HbA1c (%) | 6.2 (0.46) | 6.1 (0.4) | 0.55 (0.35, 0.86) | 0.01 |
| HbA1c (mmol/mol) | 43.8 (5.0) | 42.7 (4.3) | 0.95 (0.91, 0.99) | 0.01 |
| Total cholesterol (mmol/l) | 5.1 (1.0) | 5.0 (1.0) | 0.90 (0.75, 1.07) | 0.21 |
| HDL cholesterol (mmol/l) | 1.4 (0.5) | 1.3 (0.5) | 0.92 (0.57, 1.50) | 0.74 |
| LDL cholesterol (mmol/l) | 3.0 (0.9) | 3.0 (0.9) | 1.01 (0.82, 1.23) | 0.95 |
| Triglycerides (mmol/l) | 1.9 (1.0) | 1.6 (0.8) | 0.76 (0.62, 0.93) | 0.01 |
| Systolic blood pressure (mmHg) | 147.2 (22.9) | 148.5 (18.8) | 1.00 (0.99, 1.01) | 0.56 |
| Diastolic blood pressure (mmHg) | 86.3 (11.9) | 86.9 (10.1) | 1.00 (0.99, 1.02) | 0.64 |
| Heart rate (bmp) | 69.7 (13.5) | 67.3 (12.7) | 0.99 (0.97, 1.01) | 0.17 |
| Weight (kg) | 90.0 (17.2) | 89.8 (16.1) | 1.00 (0.99, 1.01) | 0.90 |
| BMI (kg/m^2^) | 32.6 (5.4) | 31.4 (5.0) | 0.96 (0.93, 0.98) | 0.002 |
| Waist circumference (cm) | 107.9 (12.9) | 108.1 (11.9) | 1.00 (0.99, 1.02) | 0.90 |
| Average steps per day | 6032.9 (3011.4 | 6217.6 (2616.0) | 1.00 (0.99, 1.00) | 0.54 |
