# Supplementary material for: Engagement, Retention, and Progression to Type 2 Diabetes: A Retrospective Analysis of the Cluster-Randomised "Let's Prevent Diabetes" Trial
Source: PLoS Med. 2016 Jul 12;13(7):e1002078. doi: 10.1371/journal.pmed.1002078 (PMC4942137; doi:10.1371/journal.pmed.1002078)
Supplement: S3 Table — (DOCX) [file pmed.1002078.s004.docx]

**S3 Table Secondary outcomes in of those who attended the core programme plus one or more refresher sessions (plus min one group) versus non-engagers and those who attended only the core session**

Coefficients show the mean effect of the intervention compared to standard care adjusted for baseline value and cluster. Adjusted columns are additionally adjusted for age, sex, deprivation score, smoking status and BMI.

|  | Mean change from baseline to three years (SD) | | | Plus min one versus SC | | Plus min one versus Non Plus min one | |
| --- | --- | --- | --- | --- | --- | --- | --- |
|  | Plus min one | Non Plus min one | Standard care | Unadjusted | Adjusted | Unadjusted | Adjusted |
| Fasting glucose | 0.01 (0.96) | 0.30 (0.96) | 0.16 (0.64) | -0.15 (-0.25, -0.04)** | -0.14 (-0.25, -0.04)** | -0.29 (-0.45, -0.13)*** | -0.24 (-0.39, -0.09)** |
| 2-hour glucose | -1.10 (2.18) | -0.14 (2.81) | -0.71 (2.45) | -0.55 (-0.74, -0.37)*** | -0.39 (-0.55, -0.22)*** | -0.99 (-1.50, -0.48)*** | -0.94 (-1.42, -0.46)*** |
| Hba1c (%) | -0.10 (0.35) | 0.004 (0.46) | 0.01 (0.44) | -0.12 (-0.23, -0.01)* | -0.12 (-0.23, -0.01)* | -0.13 (-0.20, -0.06)*** | -0.14 (-0.22, -0.06)** |
| Total Cholesterol (mmol/l) | -0.25 (0.86) | -0.33 (0.81) | -0.18 (0.90) | -0.11 (-0.24, 0.03) | -0.09 (-0.22, 0.04) | 0.01 (-0.18, 0.20) | 0.05 (-0.11, 0.21) |
| HDL cholesterol (mmol/l) | 0.02 (0.42) | 0.03 (0.33) | 0.02 (0.46) | -0.02 (-0.09, 0.05) | -0.03 (-0.10, 0.03) | -0.03 (-0.08, 0.02) | -0.03 (-0.09, 0.04) |
| LDL cholesterol (mmol/l) | -0.32 (0.72) | -0.34 (0.66) | -0.24 (0.78) | -0.10 (-0.20, 0.01) | -0.08 (-0.19, 0.03) | -0.02 (-0.18, 0.15) | 0.02 (-0.13, 0.17) |
| Triglyceride (mmol/l) | -0.03 (0.72) | -0.09 (0.84) | 0.02 (0.80) | -0.07 (-0.18, 0.03) | -0.05 (-0.16, 0.06) | -0.01 (-0.15, 0.14) | 0.01 (-0.12, 0.14) |
| Body weight (kg) | -0.97 (4.74) | 0.32 (4.08) | -0.46 (5.02) | -0.78 (-1.78, 0.23) | -0.77 (-1.79, 0.25) | -1.57 (-2.62, -0.52)** | -1.21 (-2.25, -0.17)* |
| BMI (kg/m^2^) | -0.29 (1.73) | 0.12 (1.47) | -0.17 (1.77) | -0.23 (-0.59, 0.13) | -0.23 (-0.60, 0.14) | -0.50 (-0.86, -0.15)** | -0.38 (-0.76, -0.01)* |
| Waist circumference (cm) | -4.33 (5.48) | -1.98 (6.22) | -3.13 (6.32) | -1.35 (-2.34, -0.47)** | -1.20 (-1.97, -0.44)** | -2.15 (-3.53, -0.78)** | -1.60 (-2.85, -0.36)* |
| Systolic BP (mmHg) | -8.11 (16.07) | -6.30 (18.35) | -8.00 (17.36) | 0.12 (-2.84, 3.09) | -0.34 (-3.23, 2.56) | -2.28 (-5.28, 0.71) | -3.21 (-6.40, -0.02)* |
| Diastolic BP (mmHg) | -4.23 (10.06) | -1.81 (9.22) | -2.50 (10.92) | -1.18 (-2.93, 0.58) | -1.34 (-3.11, 0.44) | -2.27 (-4.39, -0.14)* | -1.90 (-4.16, 0.35) |
| Heart rate (bpm) | -0.97 (9.48) | -0.07 (10.60) | -0.63 (10.12) | -0.90 (-2.25, 0.45) | -0.62 (-2.00, 0.76) | -1.34 (-3.29, 0.62) | -0.74 (-2.84, 1.37) |
| Anxiety score | -0.53 (2.81) | -0.88 (2.86) | -0.38 (2.65) | -0.11 (-0.54, 0.33) | -0.12 (-0.56, 0.33) | 0.05 (-0.71, 0.81) | 0.03 (-0.75, 0.81) |
| Depression score | 0.05 (2.34) | -0.24 (2.49) | 0.03 (2.14) | 0.03 (-0.35, 0.42) | 0.01 (-0.39, 0.42) | 0.24 (-0.24, 0.72) | 0.24 (-0.30, 0.78) |
| Quality of life | -0.003 (0.05) | 0.01 (0.07) | -0.02 (0.06) | 0.01 (0.002, 0.03)* | 0.02 (0.002, 0.03)* | -0.01 (-0.03, 0.01) | -0.01 (-0.03, 0.01) |
| Sitting time (mins) | -25.04 (162.85) | -50.83 (207.65) | -6.53 (170.27) | -22.25 (-45.43, 0.93) | -22.91 (-45.73, -0.10)* | 2.35 (-32.12, 36.82) | 11.86 (-25.81, 49.53) |
| Average steps | -190.11 (2752.47) | -528.40 (2920.87) | -776.14 (2740.46) | 607.66 (-27.06, 1242.39) | 467.15 (-157.72, 1092.03) | 274.32 (-582.70, 1131.34) | 10.22 (-617.80, 638.22) |

*p<0.05, **p<0.01, ***p<0.0001
